# The Genotypes/Subtypes and Antiviral Drug Resistance of the Hepatitis C Virus from Patients in a Tertiary Care Hospital in Nepal

**DOI:** 10.3390/v17030377

**Published:** 2025-03-06

**Authors:** Hari Prasad Kattel, Sangita Sharma, Kristian Alfsnes, John H.-O. Pettersson, Rahul Pathak, Serina Beate Engebretsen, Komal Raj Rijal, Prakash Ghimire, Åshild K. Andreassen, Megha Raj Banjara

**Affiliations:** 1 Central Department of Microbiology, Tribhuvan University, Kathmandu 44601, Nepal; hariprasadkattel@gmail.com (H.P.K.); komal.rijal@cdmi.tu.edu.np (K.R.R.); prakash.ghimire@cdmi.tu.edu.np (P.G.); 2 Tribhuvan University Teaching Hospital, Kathmandu 44601, Nepal; sangitaazz123@gmail.com (S.S.); pathak.drrahul@gmail.com (R.P.); 3 Norwegian Institute of Public Health, P.O. Box 222, NO-0213 Oslo, Norway; kristian.alfsnes@fhi.no (K.A.); serinabeate.engebretsen@fhi.no (S.B.E.); ashildkristine.andreassen@fhi.no (Å.K.A.); 4 Zoonosis Science Center, Clinical Microbiology, Department of Medical Sciences, Uppsala University, SE-751 05 Uppsala, Sweden; john.pettersson@uu.se; 5 Department of Microbiology, Swedish Veterinary Agency, SE-751 05 Uppsala, Sweden; 6 Department of Microbiology and Immunology, Peter Doherty Institute for Infection and Immunity, University of Melbourne, Melbourne, VIC 3121, Australia

**Keywords:** hepatitis C virus, subtype, antiviral drugs, resistant mutations, Nepal

## Abstract

While direct-acting antivirals (DAAs) are available for the treatment of chronic Hepatitis C virus (HCV) patients in Nepal, knowledge of the circulating genotypes/subtypes and drug target gene mutations of HCV is currently unavailable. Here, we describe HCV genotypes/subtypes and identify antiviral target gene mutations in patients at a tertiary care hospital using genome data. A cross-sectional study was conducted from December 2019 to February 2024, where PCR followed by whole genome sequencing was performed to identify HCV genotypes/subtypes and drug target gene mutations. Among all the patients who tested positive for anti-HCV, 70.6% (149/211) were HCV RNA positive, while 68.2% (30/44) were genotype/subtype 3a, followed by 1a (18.2%, 8/44) and others (13.6%, 6/44), including new subtypes 3g and 3i from Nepal. Subtype 3a was also the dominant subtype (≥70%) among intravenous drug users and sexual routes of transmission. We found 70.5% of the samples with resistant mutations in the NS3/4A region, 22.7% in NS5A, and 45.5% in NS5B. Resistant mutations against sofosbuvir, pibrentasvir, velpatasvir, daclatasvir, and dasabuvir were found at 25%, 18%, 16%, 16%, and 2%, respectively, mostly on subtype 3a. The predominant HCV genotype/subtype in our patient group was 3a, and resistance mutations against direct-acting antivirals were found in most untreated patients.

## 1. Introduction

Globally, 50 million people are living with chronic Hepatitis C virus (HCV) infection. The estimated HCV incidence was one million in 2022 with 242,000 HCV-related deaths, mostly from liver cirrhosis and liver cancer [1]. In Nepal, around 130,000 people have been chronically infected with HCV [2].

HCV has been classified into eight genotypes and >90 subtypes [3,4]. Genotype 1 is the most common worldwide, comprising almost 50% of the HCV-infected population [5]. In Nepal, genotype 3 is the predominant one, followed by genotype 1 [6,7,8].

No effective vaccine has been developed against HCV yet. The use of direct-acting antivirals (DAAs) has become more widespread for the treatment of HCV patients. DAAs comprise various groups of drugs that act as protein inhibitors that inhibit the NS3/4A protease, the NS5A replication complex, and the NS5B polymerase, which are essential for the replication of HCV. Currently, sofosbuvir, which acts on the NS5B polymerase, combined with velpatasvir, ledispasvir, or daclatasvir acts on NS5A replication complexes, and it is more commonly used in practice [9]. Considering the high cost and unavailability of genotype testing facilities, particularly in low-income countries, WHO has recommended a combination of these drugs, covering all HCV genotypes without any prior HCV genotype information, to treat chronically infected patients [1]. Although DAAs are supposed to have a success rate of more than 95% for HCV-infected patients, clinical studies of these DAAs were based on genotypes/subtypes that were prevalent in high-income countries [10]. Furthermore, virus mutations in the drug target sites may lead to the development of resistance to these DAAs as well [11]. Drug-resistant mutations can be overcome by using a combination of drugs from different classes of medications.

Although DAA drugs are available for the treatment of chronic HCV patients, few studies have been performed globally, particularly in low-income countries, using whole genome sequences to characterize genotypes/subtypes of HCV and its drug-resistant status. To improve this knowledge gap, our study aimed to describe HCV genotypes/subtypes and identify antiviral target HCV gene mutations in patients at a tertiary care hospital in Nepal. This knowledge is essential to ensure the effective treatment of patients and for worldwide disease control.

## 2. Materials and Methods

### 2.1. Study Design and Population

This was a hospital-based cross-sectional study conducted at Tribhuvan University Teaching Hospital (TUTH), Kathmandu, Nepal. A total of 211 HCV antibody-positive patients were included in this study. All patients were confirmed to be treatment-naïve based on personal interviews and an examination of past and present medical reports. Table 1 describes the demographic characteristics of the patients included in the study.

### 2.2. Sample Size and Data Collection Period

The patient sample inclusion period was from December 2019 to February 2024. We screened a total of 25,133 patients with clinically suspected HCV infection. Those cases were identified by physicians based on the clinical history of the patient, risk behaviors, and as routine tests for surgical and medical procedures including donated blood and hemodialysis. The molecular work of this study, including whole genome sequencing of 44 HCV RNA samples, was carried out at TUTH and the Norwegian Institute of Public Health, Norway, respectively. The samples were exclusively selected based on lower Ct-values (≤26), without any regard to demographic data.

### 2.3. Detection of Hepatitis C Virus Infections and Data Collection

Serological tests were performed using enzyme-linked immunosorbent assays to detect total antibodies against HCV (Autobio Diagnostics Co., Ltd., Zhengzhou, China) in the patient’s serum according to the manufacturer’s instructions. A semi-structured questionnaire was completed with the hepatitis C seropositive patients, exploring demographic characteristics, risk behaviors, and the routes of transmission.

### 2.4. HCV RNA Extraction and Detection

RNA was extracted from a 140 µL serum sample using the QIAamp viral RNA mini kit protocol (Qiagen, Hilden, Germany). Finally, 60 µL of eluted RNA was obtained and kept at −80 °C. In-house, nested reverse-transcription PCR was used to detect HCV RNA [12], and the qPCR method was used for HCV RNA detection as well as Ct value measurement via the QuantiTect Virus Kit (Qiagen, Hilden, Germany).

### 2.5. Sequencing of HCV RNA

Whole genome sequencing of HCV RNA was performed on the Illumina platform. Libraries were prepared following the protocol of the KAPA RNA Hyperprep kit (Roche, Pleasanton, CA, USA), using 18 cycles in the library amplification step. Capture-based viral enrichment was performed using the VirCapSeq-VERT probe panel following the protocol of SeqCap EZ Hypercap (Roche, Pleasanton, CA, USA) [13]. DNA was quantified by using the Qubit dsDNA HS kit (Thermo Fisher Scientific, Waltham, MA, USA), and DNA fragment size was measured with the Tapestation D1000 protocol (Agilent Technologies, Waldbronn, Germany). The final sequencing sample pool was prepared and loaded on the Illumina MiSeq instrument (Illumina Inc., San Diego, CA, USA) following the procedure of MiSeq sequencing (2 × 300 bp kit) and the MiSeq System User Guide (Illumina Inc., San Diego, CA, USA). The retrieved sequences from MiSeq were processed using a species-specific pipeline developed at NIPH. Briefly, the reads were trimmed using TrimGalore version 0.6.10 with settings q 30 and length 50 parameters (https://github.com/FelixKrueger/TrimGalore/, accessed on 29 June 2024). The trimmed reads were first cleaned, edited, and mapped to the HCV genome reference database consisting of more than 200 subtypes (Supplementary Material Table S1) using the Tanoti mapper version 9.1 with a mapping stringency of 85 (https://github.com/vbsreenu/Tanoti, accessed on 29 June 2024). The reference with the most mapped reads was selected as the “major genotype”, and all trimmed reads were mapped again to this mapper using Tanoti with a stringency of 95. Duplicate reads were removed using samtools, and consensus sequences were created with bcftools. Genome positions with coverage < 6 were masked with an “N”. Using a specific NGS script, developed at NIPH, resulted in relevant sequencing data such as HCV sub/genotype, % coverage, n mapped reads, and n duplicates. The generated HCV sub/genotype bam files were sent to the online software HCV GLUE (http://hcv-glue.cvr.gla.ac.uk/#/home, accessed on 29 June 2024) to generate DAA drug resistance reports containing resistance associate substitutions (RASs). The whole genome sequences of 44 HCVs were submitted to NCBI GenBank and published with accession numbers PQ365497–PQ365540.

### 2.6. Phylogenetic Analysis

After the NCBI online blast of our sequences, we identified the most similar publicly available HCV whole genome sequences in the NCBI GenBank database. These sequences were then used to build phylogenetic trees. All reference whole genome sequences were retrieved from GenBank in November 2024. All sequences were aligned by muscle, and the maximum likelihood phylogenetic trees were constructed with the general time reversible (GTR) model with bootstrap replicates using the MEGA version 11 software [14]. The trees were then annotated in FigTree version 1.4.4 (https://github.com/rambaut/figtree, accessed on 7 January 2025).

### 2.7. Data Management and Analysis

The prevalence of genotypes/subtypes of hepatitis C cases was calculated using a Microsoft Excel spreadsheet and SPSS version 25. Similarly, the distribution of genotypes/subtypes and drug-resistant mutations was described based on demographic and risk factors for hepatitis C. A chi-square test of independence was applied to explore the relationship between HCV genotypes/subtypes and demographic characteristics; a *p*-value ≤ 0.05 was regarded as statistically significant.

## 3. Results

### 3.1. HCV RNA Results and Genotype/Subtype Distribution of HCV

Out of the 25,133 patients with suspected HCV infection, 211 (0.8%) were anti-HCV positive, of which 70.6% (149/211) were HCV RNA confirmed via PCR. Among the forty-four HCV RNA samples analyzed via whole genome sequencing, 68.2% (thirty) were subtype 3a, followed by 1a (18.2%, eight) and 1b, 3b, 3d, 3e, 3g, and 3i (2.3%, one each).

### 3.2. Genotype Distribution According to Demographic Variables of HCV Patients

The predominant HCV genotype and subtype was found to be 3a followed by 1a and others (*p* < 0.0001) (Table 2). No significant association was found between HCV genotypes and gender (*p* = 0.9786), caste (*p* = 0.4887), age group (*p* = 0.3129), religion (*p* = 0.7916), alcohol intake habits (*p* = 0.6471), and smoking (*p* = 0.9571) (Table 2).

### 3.3. Genotype/Subtype Distribution of HCV Among Self-Reported IV Drug Use and Sexual Route of Transmission

Among the various self-reported routes of HCV transmission, the predominant routes were intravenous (IV) drug use, followed by sexual contact and others. In both routes of HCV transmission, the distribution pattern of the subtypes was found to be statistically insignificant (*p =* 0.8715). Despite this, genotype 1b was detected in one patient linked to IV drug use, whereas genotype 3e was detected in one patient linked to the sexual route of HCV transmission.

### 3.4. Phylogenetic Analysis of HCV Genomes from Nepal

**Figure 1:**
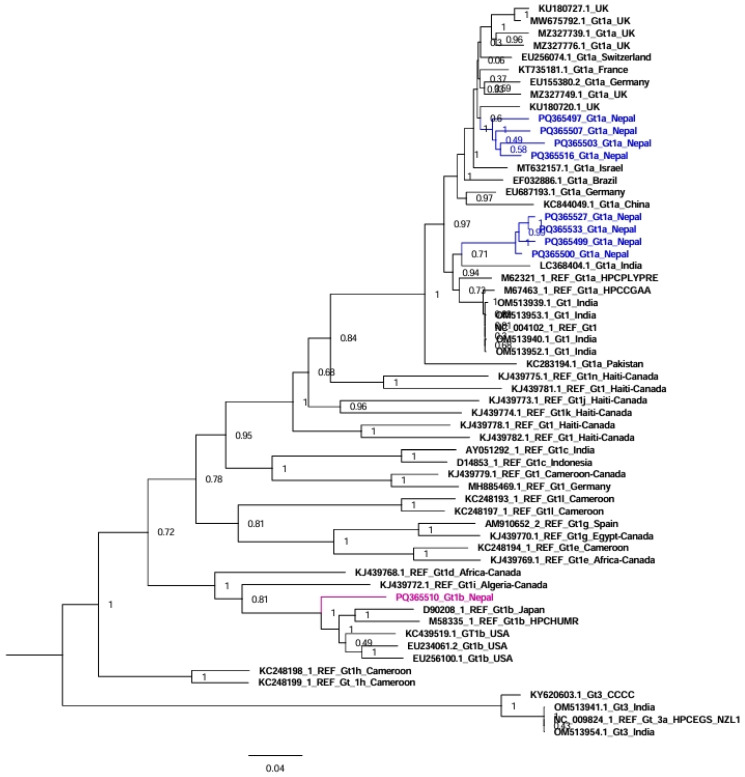
Phylogenetic tree of Nepalese HCV genotype/subtype 1 genome sequences compared to representative genome sequences from various countries (including neighboring countries like India and China). In the HCV genotype 1 tree, genotype 3 sequences were used as an outgroup. Bootstrap values to indicate support for the clustering of sequences are displayed on the nodes. This analysis involved 60 whole genome sequences for HCV genotype 1.

In the tree analysis of genotype/subtype 1, nine sequences from Nepal together with fifty-one reference sequences were used in the tree analysis (Figure 1). Here, subtype 1a sequences from Nepal clustered separately with sequences from the UK and India (four and four sequences, respectively), while subtype 1b sequences clustered with sequences from the USA and Japan (Figure 1).

**Figure 2:**
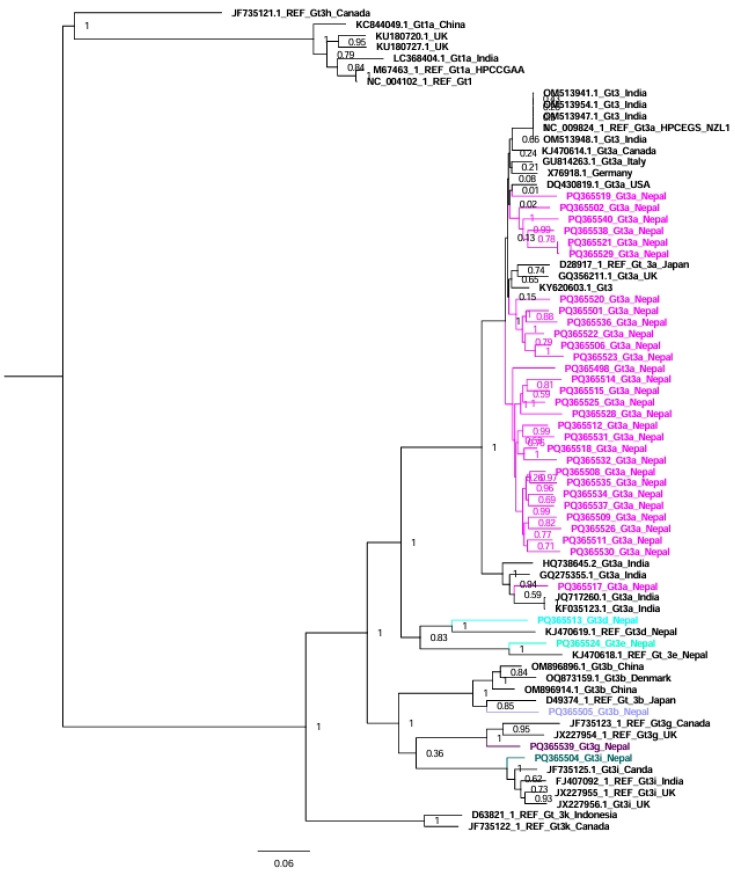
Phylogenetic tree of Nepalese HCV genotype/subtype 3 genome sequences compared to representative genome sequences from various countries (including neighboring countries like India and China). In the HCV genotype 3 tree, genotype 1 sequences were used as an outgroup. Bootstrap values to indicate support for the clustering of sequences are displayed on the nodes. This analysis involved 72 whole genome sequences for HCV genotype 3.

For genotype/subtype 3, 35 sequences from Nepal in addition to 37 reference sequences were used in the tree analysis (Figure 2). Similar to genotype 1, the sequences of HCV genotype 3 clustered with sequences from the UK, Japan, the USA, Canada, Germany, Italy, New Zealand, and India (30 sequences), while subtype 3b clustered with sequences from Japan; subtype 3d and 3e sequences clustered with previously published sequences from Nepal; subtype 3g sequence clustered with sequences from Canada and the UK; and subtype 3i sequence clustered with sequences from Canada (Figure 2).

### 3.5. Polymorphisms and Resistant Mutations on the Direct-Acting Antiviral Drug Target Site of the HCV Genome

**Figure 3:**
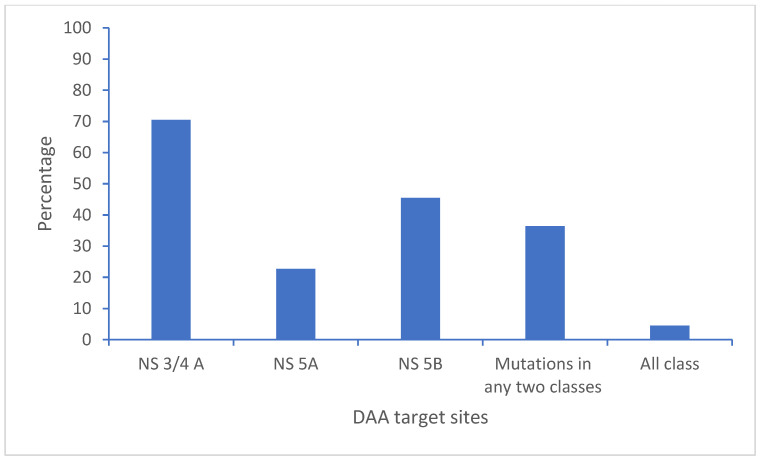
Polymorphisms and resistant mutations on the direct-acting antiviral drug target site of the HCV genome (N = 44).

Among the 44 HCV genome sequences from Nepal, DAA-resistant mutations were found in 37 genome sequences, with the highest in the NS3/4A region of HCV (70.5%) of the sequences, followed by 22.7% in NS5A and 45.5% in NS5B. Similarly, 36.4% of the sequences had resistant mutations on more than one region of the HCV genome. Two genome sequences (4.5%) had resistant mutations in all three regions (Figure 3).

In the non-structural protein NS3/4A region of HCV, we detected the following substitutions: A166S, A166T, K/Q80K, I132I, A156A, D168D, I170V, L132I, I/V170I, V55A, Y56Y, Q168Q, and I170I. The most frequent amino acid substitution mutations were detected on the targeting sites for the DAA drug grazoprevir, followed by the DAA drugs paritaprevir, voxilaprevir, and glecaprevir (Table 3).

In the non-structural protein NS5A region of HCV, L28I, F/L37L, A30K, K/R30K, K30K, M/V31M, Y93H, A30L, Y93H, P58T, and M31M substitutions were detected. The most frequent amino acid substitution mutations were detected on the targeting sites for the DAA drug pibrentasvir, followed by the DAA drugs elbasvir, daclatasvir, velpatasvir, and ombitasvir (Table 3).

Furthermore, at the NS5B region of HCV, amino acid substitutions were C/N316N, E/K206E, and A/T/V150V. The most frequent amino acid substitutions were detected for the DAA drug sofosbuvir, followed by the DAA drug dasabuvir (Table 3).

### 3.6. Anti-Viral Drug Susceptibility Profile for HCV

**Figure 4:**
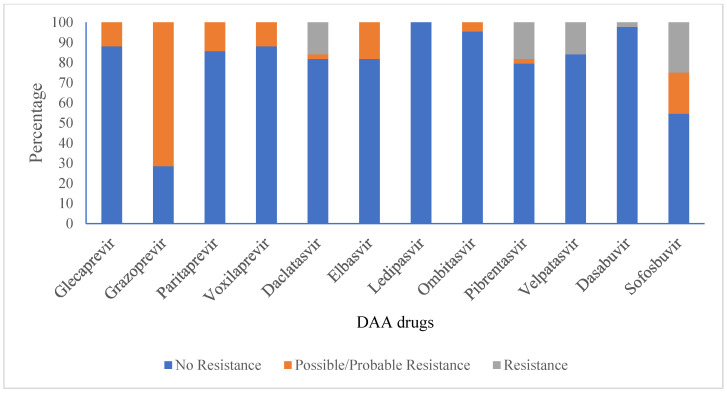
Anti-viral drug susceptibility profile of HCV (N = 44).

Viral-resistant mutations against sofosbuvir, velpatasvir, daclatasvir, and dasabuvir were 25%, 18%, 16%, 16%, and 2%, respectively, for all HCV sequences. Mutations that can probably contribute to resistance were detected for grazoprevir (71%), sofosbuvir (20%), elbasvir (18%), paritaprevir (14%), voxilaprevir (12%), glecaprevir (12%), ombitasvir (5%), daclatasvir (2%), and pibrentasvir (2%). However, there were no viral-resistant mutations found against ledipasvir (Figure 4, Table 3).

In the case of sofosbuvir resistance-associated mutation in twenty HCV genomes, three (15%) had resistant mutations to daclatasvir; two (10%) had resistant mutations to velpatasvir, pibrentasvir, and elbasvir; and one (5%) had resistant mutations to ombitasvir among NS5A protease inhibitors.

### 3.7. Anti-Viral Drug Resistance of Genotypes 3a and 1a

As the predominant subtype, for subtype 3a, viral-resistant mutations against sofosbuvir and pibrentasvir were found in 23% and 7%, respectively, of the sequenced HCV genomes. Resistance-conferring mutations were likely detected for grazoprevir (77%), sofosbuvir (30%), glecaprevir (17%), elbasvir (10%), daclatasvir (10%), velpatasvir (10%), paritaprevir (7%), ombitasvir (7%), and pibrentasvir (3%). However, there were no resistant mutations found against ledipasvir, dasabuvir, and voxilaprevir. In the case of subtype 1a, mutations that can probably contribute to resistance were detected for grazoprevir (63%), voxilaprevir (63%), and paritaprevir (50%). Resistance mutations towards ledipasvir, dasabuvir, and voxilaprevir were not detected among subtype 3a-infected patients, whereas resistance mutations against ledipasvir, sofosbuvir, elbasvir, glecaprevir, ombitasvir, daclatasvir, pibrentasvir, velpatasvir, and dasabuvir were not detected among subtype 1a. Common to both subtypes 3a and 1a, there were no viral-resistant mutations against ledipasvir and dasabuvir.

## 4. Discussion

This is a baseline genomic study on HCV in Nepal, where patient samples were collected before their treatment started. We found that the genotypes/subtypes of HCV circulating in Nepal are similar to strains circulating in neighboring countries, particularly India and other countries where there are frequent visits by Nepalese people or vice versa. Drug-resistant mutations to commonly used antivirals are in significant proportions, alarming to the control and treatment of HCV infections.

In our study, we found that genotype/subtype 3a was significantly predominant. However, we could not find significant differences in gender, age, caste, religion, alcoholic habits, and HIV-positive status. We also identified HCV subtypes 3g and 3i for the first time in Nepal. To our knowledge, a limited number of HCV genotypic studies have been conducted in Nepal, mainly based on genotype/subtype-specific PCR methods, and very few previous studies have used HCV whole genome sequencing. Our study confirms the findings from previous studies that genotype 3 is the predominant one, followed by genotype 1. Previous studies from Nepal reported subtypes 1a, 1b, 3a, 3b, 3c, 3d, 3e, and 3f in addition to 5a [6,7,8]. However, we could not detect genotypes other than 1 and 3.

Various studies from all over the world reported that subtype 1b was circulating predominantly, followed by subtype 3a and others [15,16,17,18,19,20,21,22]. However, supporting our study, genotype 3 was predominant in India, Pakistan, and Bangladesh [23,24,25,26]. Both genotypes 1 and 3 were equally predominant in China [27,28,29,30]. Our HCV genotype/subtype distribution was expected to be similar to the distribution of HCV genotype/subtype distribution in India because of closer connectivity due to cultural similarity and open border access.

Genotype 1 sequences from Nepal clustered with sequences from India, the UK, the USA, and Japan. Similarly, genotype 3 sequences from Nepal clustered with sequences from India, the UK, the USA, Canada, Germany, Italy, New Zealand, and Japan. Although both China and India are neighboring countries to Nepal, we could not find any HCV sequences from Nepal that clustered with sequences from China. Nepalese people go abroad and receive treatment for many diseases in India, including blood transfusion, hemodialysis, and other medical/surgical procedures, which might be the cause of the transmission of HCV in some patients. The sequences of subtypes 3d and 3e from Nepal were phylogenetically related to the previous sequences reported from Nepal in 1994, indicating that these subtypes are still circulating in Nepal [6].

Various classes of DAAs have become available for HCV treatment since 2014, including NS3/4A protease inhibitors (e.g., glecaprevir, voxilaprevir, grazoprevir, and paritaprevir), NS5A replication complex inhibitors (e.g., ledipasvir, velpatasvir, pibrentasvir, elbasvir, daclatasvir, and ombitasvir), and NS5B nucleoside polymerase inhibitors (e.g., sofosbuvir and dasabuvir) [31]. In Nepal, sofosbuvir together with ledispasvir or daclatasvir was started as a treatment for chronically HCV-infected patients in 2016 [32]. Currently, a combination of sofosbuvir and velpatasvir is being used for the treatment. A cohort study conducted in China has shown that treatment with sofosbuvir and velpatasvir resulted in sustained virological response (SVR) rates of 100% (16/16) after 12 weeks of treatment [33]. Although we did not assess the clinical response of HCV patients after the treatment in our study, we found that viral-resistant mutations developed on some or all DAA drug-targeting sites in some of the HCV genomes of untreated patients. Although patients were untreated with DAA drugs before enrolment in this study, they might have been infected with viruses already conferring mutations to different circulating DAAs and transmitted from other infected patients. It has also been reported that Gt3b and Gt3g subtypes inherently carry resistance substitutions [34]. Furthermore, more resistance mutations were detected in the NS3/4A region of HCV. Supporting our findings, a study showed that the baseline frequency of resistance-associated substitutions (RASs) was 37% in NS3, 29% in NS5A, and 15–24% in the NS5B region of HCV genomes [35]. A systematic review of data between 2014 and 2023 for patients who had virological failure after treatment found that the pooled prevalence of HCV resistance-associated substitutions (RASs) was 78.0% for sofosbuvir/velpatasvir, 81.0% for sofosbuvir/daclatasvir, and 79.0% for glecaprevir/pibrentasvir, and a high percentage of resistance was detected in the NS5A region of HCV [36].

The amino acid substitutions 80K, 170V, and 168Q in NS3, 93H, 31M in NS5A, as well as 316N in the NS5B regions found in our study, were similar to the results from Italy [37]. Similarly, along with the Q80K/R amino acid substitution, a study carried out on HCV3a-infected patients at tertiary care hospitals in Pakistan detected other amino acid substitutions such as L36P, Q41H, A156Y, and Q168R mutations in the NS3 region and L159F and C316R mutations in the NS5B region [38].

In our study, we could not detect the resistance amino acid substitution for ledipasvir. A systematic review and meta-analysis study to assess the efficacy and safety of ledipasvir/sofosbuvir for hepatitis C among drug users reported an 89.8% pooled SVR rate [39]. Therefore, ledipasvir-incorporated combination drugs could be effectively used as an alternative to cure HCV-infected patients. However, we did not follow up with the patients to investigate the treatment course and SVR rate since our study focused on genotypes and baseline resistance mutations.

In our findings, we detected resistance associated with amino acid substitution in many antiviral drugs among HCV genotype/subtype 3a compared to genotype/subtype 1a, which could be due to the sample size difference between these subtypes. We could not detect any resistance amino acid substitution in both the NS5A and NS5B regions of HCV genotype 1a. A study carried out on Brazilian patients detected more amino acid substitution in the NS3 and NS5A regions in HCV genotype 3a than in HCV genotype 1 [40].

Not only did we detect HCV variants harboring many types of amino acid substitutions associated with resistance to DAA, but we also found that most of the patients were either sofosbuvir-resistant or velpatasvir-resistant, not both. Since the mutation process is ongoing for HCV, HCV may develop known and probable resistance mutations to available DAAs in the future. This may influence the treatment regime in Nepal. Knowledge of previous resistance, treatment with combination drugs, and the prevention of transmission through vaccine development or other measures are important for reducing the burden of the hepatitis C virus.

## 5. Conclusions

The most predominant HCV genotype/subtype was 3a in HCV-infected patients in Nepal. New HCV subtypes 3g and 3i were detected for the first time in Nepal. Direct-acting antiviral drug resistance-associated mutations are present in HCV patients in Nepal. Since we could not detect any resistant amino acid substitutions for ledipasvir, ledipasvir/sofosbuvir combination drugs could be used as another alternative for the treatment of chronically HCV-infected patients. However, other antiviral drug-resistant genotypes are present, these need to be taken into consideration before treatment is started. The regular surveillance of genotypes/subtypes and drug target gene mutations could help in the appropriate treatment and control of hepatitis C in Nepal.

## Figures and Tables

**Table 1:** Demographic description of the study population (N = 211).

Variables		Number (N)	Percentage
Gender	Male	174	82.5
	Female	37	17.5
Caste	Janajati	105	49.8
	Brahmin/Chhetri	69	32.7
	Madeshi	19	9.0
	Dalit	14	6.6
	Thakuri	4	1.9
Age	Pediatric group (0–14 yrs)	1	0.5
	Young group (15–47 yrs)	156	73.9
	Middle age group (48–63 yrs)	48	22.7
	Elderly group (above 64 yrs)	6	2.8
Religion	Hindu	163	77.3
	Buddhist	25	11.8
	Christian	18	8.5
	Islam	3	1.4
	Kirat	2	0.9
Alcohol intake	Non-alcoholic	141	66.8
	Regular alcoholic	70	33.2
Smoking	Regular smoker	135	64.0
	Non-smoker	76	36.0
Co-infected with HIV	Non-infected	197	93.4
	Co-infected	14	6.6
Self-reported route of transmission	IV drug use	96	45.5
	Sexual	59	28.0
	Unknown	24	11.4
	Blood transfusion	13	6.2
	Haemodialysis	9	4.3
	Professional exposure	3	1.4
	Tattooing	2	0.9
	Blood transfusion or tattooing	2	0.9
	Tattooing or sexual	2	0.9
	Blood transfusion or hemodialysis	1	0.5

**Table 2:** Genotype distribution according to the demographic characteristics of HCV patients.

Variables	Genotype 1 (1a, 1b) N (%)	Genotype 3 (3a, 3b, 3d, 3e, 3g, 3i) N (%)	*p*-Value
*Gender*			
Male	8 (20.5)	31 (79.5)	0.9786
Female	1 (20)	4 (80)	
*Caste*			
Brahmin/Chhetri	6 (28.6)	15 (71.4)	0.4887
Dalit	1 (20)	4 (80)	
Janajati	1 (7.1)	13 (92.9)	
Madeshi	1 (25)	3 (75)	
*Age group*			
Pediatric (0–14 yrs)	-	1 (100)	
Young (15–47 yrs)	8 (25)	24 (75)	0.3129
Middle age (48–63 yrs)	1 (10)	9 (90)	
Elderly (Above 64 yrs)	-	1 (100)	
*Religion*			
Buddhist	1 (14.3)	6 (85.7)	
Christian	1 (33.3)	2 (66.7)	
Hindu	7 (21.2)	26 (78.8)	0.7916
Kirat	-	1 (100)	
*Alcohol intake*			
Non-alcoholic	6 (21.2)	26 (78.8)	0.6471
Regular alcoholic	3 (25)	9 (75)	
*Smoking*			
Non-smoker	3 (20)	12 (80)	
Regular smoker	6 (20.7)	23 (79.3)	0.9571
*Co-infected with HIV*			
Co-infected	-	3 (100)	
Non-infected	9 (21.9)	32 (78.1)	NA *

* Not applicable due to one of the cell values containing zero.

**Table 3:** Type of polymorphisms and resistant mutations on direct-acting antiviral drug target sites of the HCV genome.

Antiviral Drug	Polymorphism/Mutation Resistant Associated Substitution (RAS)	Genotype/Subtype	Resistance Type (N) *	
	*NS3/4A Protein*		Category II, III	Category I
Glecaprevir	A166S	3a	2	
	A166T	3a	3	
Grazoprevir	K/Q80K; K/Q80K + I132I; K/Q80K + A156A; K/Q80K + D168D	1a	3	
	K/Q80K; K/Q80K + I132I; K/Q80K + A156A; K/Q80K + D168D; I170V	1a	1	
	L132I + I/V170I	3a	1	
	V55A; I170V	1a	1	
	Y56Y + Q168Q + I/V170I	3a	18	
	Y56Y + Q168Q + I/V170I; L132I + I/V170I	3a	4	
	Y56Y + Q168Q + I170I	3b, 3d	2 (1 each)	
Paritaprevir	A166S	3a	2	
	K/Q80K	1a	4	
Voxilaprevir	K/Q80K	1a	4	
	V55A	1a	1	
	*NS5A Protein*			
Daclatasvir	L28I; F/L37L	1b	1	
	A30K	3a		1
	K/R30K	3i		1
	K30K	3d, 3e		2 (1 each)
	K30K; K30K + M/V31M; M/V31M	3g		1
	Y93H	3a		2
Elbasvir	A30K	3a	1	
	A30L; Y93H	3a	1	
	K/R30K	3i	1	
	K30K	3b, 3d, 3e	3 (1 each)	
	K30K; K30K + M/V31M	3g	1	
	Y93H	3a	1	
Ledipasvir	Not found			
Ombitasvir	Y93H	3a	1	
Pibrentasvir	P58T	3a	1	
	A30K	3a		1
	K/R30K	3i		1
	K30K	3d, 3e		2 (1 each)
	K30K; M/V31M; K30K + M/V31M	3g		1
	K30K; M31M	3b		1
	Y93H	3a		2
Velpatasvir	A30K	3a		1
	K/R30K	3i		1
	K30K	3d, 3e		2 (1 each)
	K30K; K30K + M/V31M; M/V31M	3g		1
	Y93H	3a		2
	*NS5B Protein*			
Dasabuvir	C/N316N	1b		1
Sofosbuvir	E/K206E	3a	9	
	A/T/V150V	3a		9
	A/T/V150V; A/T/V150V + E/K206E; E/K206E	3a		1
	C/N316N	1b		1

* Resistance: Category I: findings have the strongest evidence: either (a) in vitro resistance level ≥ 5 and found at baseline or treatment-emergent in vivo, or (b) both found at baseline and treatment-emergent. Probable resistance: Category II: in vitro level ≥ 5, found at baseline, or treatment-emergent. Category III: in vitro level ≥ 5.

## Data Availability

All data has already been provided in the article. Sequence data are available in GenBank.

## Supplementary Materials

The following supporting information can be downloaded at https://www.mdpi.com/article/10.3390/v17030377/s1, Table S1: HCV genome reference database.
