# Supplementary material for: The Genotypes/Subtypes and Antiviral Drug Resistance of the Hepatitis C Virus from Patients in a Tertiary Care Hospital in Nepal
Source: Viruses. 2025 Mar 6;17(3):377. doi: 10.3390/v17030377 (PMC11946309; doi:10.3390/v17030377)
Supplement: Supplementary file 1 [file viruses-17-00377-s001.zip › viruses-3509914-supplementary.pdf]

HCV genome reference database (Supplementary Material Table S1)

Accession number	Genotype	Subtype	Country of origin	Date of sampling
AF009606	1	1a		Before 19-JUN-1997
M62321	1	1a		Before 1991
M67463	1	1a		Before 1991
HQ850279	1	1a	USA	Before 06-JAN-2011
EF407457	1	1a	African-american	Before 29-JAN-2007
D90208	1	1b	Japan	Before 1990
M58335	1	1b		Before 1991
EU781827	1	1b		Before 29-MAY-2008
EU781828	1	1b		Before 29-MAY-2008
D14853	1	1c	Indonesia	Before 01-APR-1993
AY051292	1	1c	India	Before 16-AUG-2001
AY651061	1	1c	India	Before 11-JUN-2004
KJ439768	1	1d	Canada	Before 12-FEB-2014
KC248194	1	1e	United Kingdom	Before 30-NOV-2012
AM910652	1	1g	Spain	Before 20-NOV-2007
KC248198	1	1h	Cameroon	Before 30-NOV-2012
KC248199	1	1h	Cameroon	Before 30-NOV-2012
KJ439772	1	1i	Canada	Before 12-FEB-2014
KJ439773	1	1j	Canada	Before 12-FEB-2014
KJ439774	1	1k	Canada	Before 12-FEB-2014
KC248193	1	1l	Cameroon	Before 30-NOV-2012
KC248197	1	1l	Cameroon	Before 30-NOV-2012
KC248196	1	1l	United Kingdom	Before 30-NOV-2012
KJ439778	1	1m	Canada	Before 12-FEB-2014
KJ439782	1	1m	Canada	Before 12-FEB-2014
KJ439781	1	1n	Canada	Before 12-FEB-2014
KJ439775	1	1n	Canada	Before 12-FEB-2014
KJ439779	1	1o	Canada	Before 12-FEB-2014
MH885469	1	1o	Germany	2017
HQ537007	1		Cyprus	09-MAY-2005
AJ851228	1			Before 21-OCT-2004
KC248195	1		United Kingdom	Before 30-NOV-2012
KJ439780	1		Canada	Before 12-FEB-2014
KJ439776	1		Canada	Before 12-FEB-2014
KJ439777	1		Canada	Before 12-FEB-2014
D00944	2	2a		Before 1991
AB047639	2	2a		Before 23-AUG-2000
HQ639944	2	2a	China	Before 20-NOV-2010
D10988	2	2b		Before 1992
AB030907	2	2b		Before 08-AUG-1999
AB661388	2	2b	Japan	Before 04-AUG-2011
AB661382	2	2b	Japan	Before 04-AUG-2011
D50409	2	2c		Before 24-APR-1995
JX227949	2	2c	United Kingdom	Before 20-JUN-2012
JF735114	2	2d	Canada	Before 28-MAR-2011

JF735120	2	2e	Canada	Before 28-MAR-2011
KC844042	2	2f	China	2010
KC844050	2	2f	China	2002
DQ155561	2	2i	Vietnam	Before 03-AUG-2005
HM777358	2	2j	Venezuela	09-NOV-2006
JF735113	2	2j	Canada	Before 28-MAR-2011
HM777359	2	2j	Venezuela	14-NOV-2005
AB031663	2	2k		Before 26-AUG-1999
JX227953	2	2k	United Kingdom	Before 20-JUN-2012
JF735111	2	2m	Canada	Before 28-MAR-2011
JX227967	2	2m	Canada	Before 20-JUN-2012
FN666428	2	2q	Spain	2002
FN666429	2	2q	Spain	2001
JF735115	2	2r	Canada	Before 28-MAR-2011
KC197238	2	2t	France	1995
JF735112	2	2u	Canada	Before 28-MAR-2011
JF735116	2		Canada	Before 28-MAR-2011
JF735118	2		Canada	Before 28-MAR-2011
JF735117	2		Canada	Before 28-MAR-2011
JF735119	2		Canada	Before 28-MAR-2011
JF735110	2		Canada	Before 28-MAR-2011
KC197236	2		France	1995
KC197237	2		France	2001
KC197239	2		France	2009
D17763	3	3a	New Zealand	Before 27-SEP-1993
D28917	3	3a		Before 12-MAR-1994
X76918	3	3a	Germany	Before 17-JAN-1995
JN714194	3	3a	India	11-JUN-2011
D49374	3	3b	Japan	Before 18-FEB-1995
JQ065709	3	3b	China	22-AUG-2011
KJ470619	3	3d	Nepal	Before 20-FEB-2014
KJ470618	3	3e	Nepal	Before 20-FEB-2014
JX227954	3	3g	United Kingdom	Before 20-JUN-2012
JF735123	3	3g	Canada	Before 28-MAR-2011
JF735126	3	3h	Somalia	Before 28-MAR-2011
JF735121	3	3h	Canada	Before 28-MAR-2011
FJ407092	3	3i	India	02-Jun-2002
JX227955	3	3i	United Kingdom	Before 20-JUN-2012
D63821	3	3k		Before 10-AUG-1995
JF735122	3	3k	Canada	Before 28-MAR-2011
JF735124	3		Canada	Before 28-MAR-2011
Y11604	4	4a		Before 03-JUN-1997
DQ988074	4	4a	Egypt	2006
DQ418789	4	4a	USA	Before 02-FEB-2006
FJ462435	4	4b	Canada	Before 07-NOV-2008
FJ462436	4	4c	Canada	Before 07-NOV-2008
DQ418786	4	4d	USA	Before 02-FEB-2006
FJ462437	4	4d	Canada	Before 07-NOV-2008

EU392172	4	4d		Before 09-JAN-2008)
EF589161	4	4f	France	Before 03-MAY-2007
EU392175	4	4f		Before 09-JAN-2008
EU392174	4	4f		Before 09-JAN-2008
FJ462432	4	4g	Canada	Before 07-NOV-2008
JX227971	4	4g	United Kingdom	Before 20-JUN-2012
JX227963	4	4g	United Kingdom	Before 20-JUN-2012
EU392173	4	4k		Before 09-JAN-2008
FJ462438	4	4k	Canada	Before 07-NOV-2008)
EU392171	4	4k		Before 09-JAN-2008
FJ839870	4	4l	Canada	Before 18-MAR-2009
JX227957	4	4l	United Kingdom	Before 20-JUN-2012
FJ462433	4	4m	Canada	Before 07-NOV-2008
JX227972	4	4m	United Kingdom	Before 20-JUN-2012
FJ462441	4	4n	Canada	Before 07-NOV-2008
JX227970	4	4n	United Kingdom	Before 20-JUN-2012
FJ462440	4	4o	Canada	Before 07-NOV-2008
JX227977	4	4o	United Kingdom	Before 20-JUN-2012
FJ462431	4	4p	Canada	Before 07-NOV-2008
FJ462439	4	4r	Canada	Before 07-NOV-2008
JX227976	4	4r	United Kingdom	Before 20-JUN-2012
JF735136	4	4s	Canada	Before 28-MAR-2011
FJ839869	4	4t	Canada	Before 18-MAR-2009
HQ537009	4	4v	Cyprus	13-FEB-2006
JX227959	4	4v	United Kingdom	Before 20-JUN-2012
HQ537008	4	4v	Cyprus	14-JUN-2005
JX227960	4	4v	United Kingdom	Before 20-JUN-2012
FJ025855	4	4b	Portugal	Before 14-AUG-2008
FJ025856	4	4b	Portugal	Before 14-AUG-2008
FJ025854	4	4b	Portugal	Before 14-AUG-2008
JX227964	4		United Kingdom	Before 20-JUN-2012
JF735127	4		Canada	Before 28-MAR-2011
JF735132	4		Canada	Before 28-MAR-2011
JF735131	4		Canada	Before 28-MAR-2011
JF735130	4		Canada	Before 28-MAR-2011
JF735129	4		Canada	Before 28-MAR-2011
JF735138	4		Canada	Before 28-MAR-2011
JF735135	4		Canada	Before 28-MAR-2011
JF735134	4		Canada	Before 28-MAR-2011
AF064490	5	5a		Before 09-MAY-1998
Y13184	5	5a		Before 30-SEP-1997
KT595242	5		Burkina Faso	01-JAN-2009
Y12083	6	6a		Before 25-MAR-1997
AY859526	6	6a	Hong Kong	Before 17-DEC-2004
HQ639936	6	6a	China	Before 20-NOV-2010
EU246930	6	6a	Vietnam	Before 24-OCT-2007
D84262	6	6b		Before 29-MAR-1996
EF424629	6	6c	Thailand	Before 06-FEB-2007

D84263	6	6d		Before 29-MAR-1996
DQ314805	6	6e	China	Before 02-DEC-2005
EU246932	6	6e	Vietnam	Before 24-OCT-2007
EU246931	6	6e	Vietnam	Before 24-OCT-2007
DQ835760	6	6f	Thailand	Before 23-JUN-2006
EU246936	6	6f	Thailand	Before 24-OCT-2007
D63822	6	6g		Before 10-AUG-1995
DQ314806	6	6g	Hong Kong	Before 02-DEC-2005
D84265	6	6h		Before 29-MAR-1996
DQ835770	6	6i	Thailand	Before 23-JUN-2006
DQ835762	6	6i	Thailand	Before 23-JUN-2006
DQ835769	6	6j	Thailand	Before 23-JUN-2006
DQ835761	6	6j	Thailand	Before 23-JUN-2006
D84264	6	6k		Before 29-MAR-1996
EF424628	6	6l	USA	Before 06-FEB-2007
JX183556	6	6l	Vietnam	Before 15-JUN-2012
DQ835767	6	6m	Thailand	Before 23-JUN-2006
DQ835766	6	6m	Thailand	Before 23-JUN-2006
DQ278894	6	6n	China	Before 03-NOV-2005
DQ835768	6	6n	Thailand	Before 23-JUN-2006
EU246938	6	6n	Thailand	Before 24-OCT-2007
EF424627	6	6o	Cananda	Before 06-FEB-2007
EU246934	6	6o	Vietnam	Before 24-OCT-2007
EF424626	6	6p	Canada	Before 06-FEB-2007
EF424625	6	6q	Canada	Before 06-FEB-2007
EU408328	6	6r	Canada	Before 16-JAN-2008
EU408329	6	6s	Canada	Before 16-JAN-2008
EF632071	6	6t	Vietnam	Before 24-MAY-2007
EU246939	6	6t	Vietnam	Before 24-OCT-2007
EU246940	6	6u	Vietnam	Before 24-OCT-2007
EU158186	6	6v	China	AUG-2004
EU798760	6	6v	China	Before 06-JUN-2008
EU798761	6	6v	China	Before 06-JUN-2008
DQ278892	6	6w	China	Before 03-NOV-2005
EU643834	6	6w	Taiwan	Before 15-APR-2008
EU643836	6	6w		Before 15-APR-2008
EU408330	6	6u	China	Before 16-JAN-2008
EU408332	6	6u	China	Before 16-JAN-2008
EU408331	6	6u	China	Before 16-JAN-2008
JX183552	6	6xb	Vietnam	Before 15-JUN-2012
KJ567645	6	6xb	Vietnam	Before 11-MAR-2014
KJ567651	6	6xc	Vietnam	Before 11-MAR-2014
KM252789	6	6xd	Laos	Before 31-JUL-2014
KM252790	6	6xd	Laos	Before 31-JUL-2014
KM252791	6	6xd	Laos	Before 31-JUL-2014
JX183557	6	6xe	China	Before 15-JUN-2012
KM252792	6	6xe	China	Before 31-JUL-2014
KJ567646	6	6xf	Vietnam	Before 11-MAR-2014

KJ567647	6	6xf	Vietnam	Before 11-MAR-2014
MH492361	6	6xg	Myanmar	2014
MH492360	6	6xg	Myanmar	2014
MH492362	6	6xg	Myanmar	2014
MG879000	6	6xh	China	2014
DQ278891	6	6k	China	Before 03-NOV-2005
DQ278893	6	6k	China	Before 03-NOV-2005
JX183558	6		Canada	Before 15-JUN-2012
JX183553	6		Vietnam	Before 15-JUN-2012
JX183554	6		Laos	Before 15-JUN-2012
JX183551	6		Vietnam	Before 15-JUN-2012
JX183549	6		China	Before 15-JUN-2012
JX183550	6		Canada	Before 15-JUN-2012
KJ470620	6		China	Before 19-FEB-2014
KJ470621	6		China	Before 19-FEB-2014
KJ470622	6		China	Before 19-FEB-2014
KJ470623	6		China	Before 19-FEB-2014
KJ470624	6		China	Before 19-FEB-2014
KJ470625	6		China	Before 19-FEB-2014
KC844039	6		China	2009
KC844040	6		China	2009
KJ567652	6		Vietnam	Before 11-MAR-2014
KJ567650	6		Vietnam	Before 11-MAR-2014
KJ567649	6		Vietnam	Before 11-MAR-2014
KJ567648	6		Vietnam	Before 11-MAR-2014
KJ567644	6		Vietnam	Before 11-MAR-2014
MG878999	6		China	2014
EF108306	7	7a	Canada	Before 03-MAR-2015
KX092342	7	7b	France	JAN-2010
MH590698	8	8a	Canada	14-MAR-2016
MH590699	8	8a	Canada	21-MAR-2016
MH590700	8	8a	Canada	06-SEP-2017
MH590701	8	8a	Canada	27-JAN-2015
